# Evolution and differentiation of the cybersecurity communities in three social question and answer sites: A mixed-methods analysis

**DOI:** 10.1371/journal.pone.0261954

**Published:** 2021-12-31

**Authors:** Muting Wu, Raul Aranovich, Vladimir Filkov

**Affiliations:** 1 DECAL Lab, Department of Computer Science, University of California, Davis, Davis, California, United States of America; 2 Department of Linguistics, University of California, Davis, Davis, California, United States of America; Sri Eshwar College of Engineering, INDIA

## Abstract

Cybersecurity affects us all in our daily lives. New knowledge on best practices, new vulnerabilities, and timely fixes for cybersecurity issues is growing super-linearly, and is spread across numerous, heterogeneous sources. Because of that, community contribution-based, question and answer sites have become clearinghouses for cybersecurity-related inquiries, as they have for many other topics. Historically, Stack Overflow has been the most popular platform for different kinds of technical questions, including for cybersecurity. That has been changing, however, with the advent of Security Stack Exchange, a site specifically designed for cybersecurity-related questions and answers. More recently, some cybersecurity-related subreddits of Reddit, have become hubs for cybersecurity-related questions and discussions. The availability of multiple overlapping communities has created a complex terrain to navigate for someone looking for an answer to a cybersecurity question. In this paper, we investigate how and why people choose among three prominent, overlapping, question and answer communities, for their cybersecurity knowledge needs. We aggregated data of several consecutive years of cybersecurity-related questions from Stack Overflow, Security Stack Exchange, and Reddit, and performed statistical, linguistic, and longitudinal analysis. To triangulate the results, we also conducted user surveys. We found that the user behavior across those three communities is different, in most cases. Likewise, cybersecurity-related questions asked on the three sites are different, more technical on Security Stack Exchange and Stack Overflow, and more subjective and personal on Reddit. Moreover, there appears to have been a differentiation of the communities along the same lines, accompanied by overall popularity trends suggestive of Stack Overflow’s decline and Security Stack Exchange’s rise within the cybersecurity community. Reddit is addressing the more subjective, discussion type needs of the lay community, and is growing rapidly.

## 1. Introduction

The state-of-the-art in cybersecurity knowledge is an ever-expanding horizon. New cybersecurity exploits are constantly being discovered and reported, software patches are created and released, and people learn about them and patch their own systems. This growing wealth of information is being stored in different places and is available publicly. The CVE database [1] is one of the most popular vulnerability databases, including near 160,000 publicly known cybersecurity vulnerabilities since its inception (in 1999), with more than 18,000 vulnerabilities published last year. VULDB [2] is another vulnerability database containing more than 180,000 vulnerabilities. Many companies on the cybersecurity frontier also have their own cybersecurity advisory websites listing vulnerabilities and associated patches regarding their products.

Because of the many different sources where information about cybersecurity is stored, and its high volume and longitudinal nature, finding the most current and accurate one can be a real challenge. For example, the Heartbleed Bug [3] was a serious vulnerability in the popular OpenSSL cryptographic library [4], a widely used implementation of the Transport Layer Security (TLS) protocol. It allowed attackers to steal protected information about the OpenSSL library used to secure the Internet. The vulnerability was first discovered on April 1, 2014, and publicly disclosed on April 7, 2014 [5]. A patch was published April 7 but in a different place [6]. Many cybersecurity advisory websites for the affected products, published the notifications and the update instructions several days later. 3% of HTTPS sites in the Alexa Top 1 Million remained vulnerable even two months after the disclosure [7].

Instead of directly searching for information at the source and then processing the findings, for a while now the social web has been providing marketplaces for bringing together people with questions and people with answers. Social question and answer (Q&A) sites like Stack Exchange (SE) [8] in general, Stack Overflow (SO) [9] in particular, and some subreddits of Reddit [10], have been remarkably helpful to people, providing answers in matter of minutes or faster.

The similar structures and functionalities of these sites, as well as their overlap, can present a challenge for novice users looking for information. For example, here are three cybersecurity-related questions: “Is it secure to use “require” with GET/POST data?”, “What’s the difference between MAC algorithm vs hash algorithm?”, and “Which email provider is the best in terms of security and privacy?”. Although each of them can be asked in either of the three platforms, we do not know which platform is the most suitable one for each question. The following, then, arise naturally: what type of cybersecurity-related information do people get from each site? Are the sites used interchangeably or complementarily? Which of those sites are the most popular and for what?

Our goal in this paper is to understand how and why people use the three similarly structured cybersecurity-related social Q&A sites: *Sec SO* (cybersecurity sub-parts of Stack Overflow), *Sec SE* (Security Stack Exchange site [11]), and *Sec Reddit* (cybersecurity-related Reddit subreddits), to obtain cybersecurity-related information. We chose these three social Q&A sites as they are the most popular and active ones for cybersecurity-related inquiries. Inspired by the publicly available data of questions and answers from those three sites, we gathered comprehensive multi-year historical data of Q&A pairs from those three sites, and undertook longitudinal and linguistic studies, using mixed-method analyses, to understand their usage patterns. To triangulate our findings, we also conducted a user survey across the three sites. We found the following:

- The variance in answer latency for *Sec SO* is the greatest, with some questions answered very quickly and others very slowly, some staying unanswered altogether. Questions asked in *Sec Reddit* on average have more answerers and answers.
- Most questioners in the three sites only ask one question. Answerers in *Sec SE* are the most active, and some very active answerers there have answered more than 50 questions each.
- Most questions asked on *Sec SO* and *Sec SE* are technical questions, with programming-related questions occupying a larger fraction of *Sec SO* questions. *Sec Reddit* contains not only technical questions but also more subjective questions, especially career inquiries.
- *Sec SO* has been steadily losing its popularity from 2011 to 2020. *Sec SE* had been steadily gaining popularity between 2011 and 2016, with evidence of migration to it from *Sec SO* users, and then has started losing some. The popularity of *Sec Reddit* has been booming since the second half of 2017, and in 2020, dropped down and bounced back.

In practice, our main contribution to the cybersecurity community is facilitating the match between people’s cybersecurity, and even social, needs and the available social Q&A sites. More theoretically, our work offers an under-the-hood view of social communities’ evolution and differentiation and the determining factors that may lead to community migration/prosperity/blight. Knowing this can help address any remaining needs appropriately.

Although social Q&A sites have been extensively studied before, existing work has mostly focused on the characteristics of a single community at a time, especially Stack Overflow. Very few papers have studied different communities of the same topic concurrently, and those that do have focused on differently structured sites, as we discuss in the prior work section. Contrasting those, our work is the first that we know of, to study multiple similarly structured social Q&A sites in the context of cybersecurity, with a view on dynamic community forming and community differentiation.

The rest of the paper is organized as follows. In Section 2, we provide a literature review about related work to this paper. Then in Section 3, we introduce the backgrounds regarding social Q&A sites and the virtual community of practice, and present our research questions. Following this, we describe our mixed methods in Section 4 and present our results in Section 5. Next, we discuss the implications of our work and potential threats to validity in Section 6. Finally, Section 7 presents our conclusions and directions for future work.

## 2. Background and research questions

Here, first we provide summaries of the three social question and answer (Q&A) sites on which we focus in this paper. Then, we summarize the community of practice theory, which provides the prism for our work. Finally, we derive our research questions.

### 2.1 Social Q&A sites

Before the growing popularity of social Q&A sites, expert service sites were the mainstream of Q&A sites, where experts in a specific area would answer questions. Most of those were fee-based. Launched in 2002 and ended in 2006, Google Answers [12] was one of the most famous expert service sites. There are two main downsides to the fee-based approach: that users have to pay for answers, and, given the lack of active experts, wait time can be long. In social Q&A sites, everyone can ask a question, answer a question, and rate a question/answer [13]. The core idea is to utilize the wisdom of crowds [14]. Because of that, most social Q&A sites are free to use, with some site-based reputation/points/tokens awarded for providing good questions and answers. There are hundreds of very popular Q&A sites in existence. In this paper, we focus on three most popular social Q&A sites where users ask many cybersecurity questions: Stack Overflow [9], Security Stack Exchange [11], and Reddit [10]. According to the latest Alexa Top Sites [15] that measure the traffic of millions of websites, Reddit ranked 19th globally, Stack Overflow ranked 52th, and Stack Exchange ranked 134th. (As a comparison to other social Q&A sites, Quora [16] ranked 351th, Answers [17] ranked 2890th, Yahoo! Answers [18] was completely shut down earlier this year).

Stack Overflow (SO), one of the largest and most popular social Q&A sites, is intended for professional and enthusiast programmers. Launched in 2008, SO now has around 22 million questions, 32 million answers, and more than 15 million users [19]. According to its own description, SO is all about getting answers instead of a discussion forum, so people should avoid asking questions that are primarily opinion-based, or that are likely to generate discussions rather than answers [20]. Instead, people should ask detailed questions about specific programming problems, software algorithms, coding techniques, and so on. Questions not meeting the standard might be closed or deleted. In this paper, we only focus on SO questions with a [security] tag, which we denote *Sec SO*.

Stack Exchange [8] is a network of many social Q&A sites. Started in 2008, Stack Exchange network now contains 177 websites featuring different specific topics. The six main categories of topics are technology, culture/recreation, life/arts, science, professional, and business. Stack Overflow is the most popular site which includes questions from a large variety of topics, whereas Security Stack Exchange is designed for including only cybersecurity-related questions and answers. Created in the second half of 2010, Security Stack Exchange now contains about 63 thousand questions, 111 thousand answers, and more than 210 thousand users [19]. The standard of questions and what people should ask in Security Stack Exchange is very similar to that of Stack Overflow. The only difference is the topic of the question. In Security Stack Exchange, people should only ask questions related to cybersecurity. In this paper, we treat the whole Security Stack Exchange site as a cybersecurity community, i.e. *Sec SE*.

Despite the different topics they focus on, all 177 websites, including SO, in the Stack Exchange network share the same web interface and functionality: everyone can ask questions, answer questions, and comment on either; questions are annotated with one or multiple tags (e.g., [security] for cybersecurity-related questions or [java] for java-related questions) chosen by the questioners; questions and answers are upvoted/downvoted by users with certain amounts of reputation; reputation is earned mainly by providing good questions and answers (upvoted by other users from this website) and lost by providing bad questions and answers (downvoted by other users from this website); a question might be automatically closed/deleted if high reputation users vote that it does not meet standard (e.g. off-topic or duplication) and an answer might also be automatically deleted if high reputation users vote that the answer does not match certain criteria; moderators can delete anything that doesn’t meet the site standard; each user has a profile page with personal information (can be empty if anonymity is desired), activity-related information (automatically provided, e.g., questions, answers, and comments posted by the user), and the reputation earned.

Reddit is one of the most popular sites on the Internet. It is home to thousands of communities, endless conversation, and authentic human connection [21]. Launched in 2005, Reddit now has more than 52 million daily active users, and millions of subreddits [21]. Different subreddits (e.g. r/worldnews [22], r/gaming [23], or even your own subreddits) are created by Reddit users for different purposes and each of them has their own rules.

In this paper, we focus on three most popular cybersecurity-related subreddits, in terms of the number of subscribers and text posts, where people can post/comment cybersecurity-related questions on: r/security [24], r/cybersecurity [25] and r/AskNetsec [26]. Although r/netsec [27] is another very popular cybersecurity-related subreddit, it only focuses on technical news, containing only link posts, a type of post that only contains an URL, thus unrelated to Q&A and this study. Currently, there are around 289 hundred subscribers for r/cybersecurity, 164 hundred subscribers for r/security, and 153 hundred subscribers for r/AskNetsec. Those three subreddits, belonging to *Sec Reddit*, do not have any specific rules about what should be asked there.

In Reddit, all subreddits have similar functionality as well: a user can post a text or a link, and comment on a post or on a comment; posts and comments are upvoted/downvoted by registered users; moderators of each subreddit can delete posts/comments that don’t meet the subreddit standard; each user has a profile page with personal information (only username), activity-related information (e.g. posts and comments posted by the user), and the karma point (calculated by subtracting the number of downvotes from the number of upvotes). However, different subreddits may have different restrictions or rules. For example, in some subreddits, only users with certain karma points can post/comment/vote, or in some other subreddits, only link posts are allowed to be posted.

### 2.2 Theory: Community of practice

Community of Practice (CoP) Theory is a social learning theory first proposed by Lave and Wenger in 1991 [28]. They proposed a new model that departs from the traditional cognitive approach of learning. This new model posits that cognition is social, and focuses on situated social interactions: the workplace, where skills are acquired and used, as well as the informal gatherings where novice and experts interact with each other [29]. Originally [28], a CoP was specifically defined as “A group of people from the same discipline, who improve their skills by working alongside experts and being involved in increasingly complicated tasks [29]”. After several years’ of refinement to the idea [30, 31], the CoP definition was eventually generalized by Wenger et al. to “Groups of people who share a concern, a set of problems, or a passion about a topic, and who deepen their knowledge and expertise in this area by interacting on an ongoing basis”. They also defined three key requirements for the existence of a CoP: “domain”, “community” and “practice”. “Domain” refers to the shared domain of interest which defines the membership of the CoP; “Community” refers to members engaging in joint activities and discussions, sharing information, and learning from each other; “Practice” indicates the shared repertoire of resources including experiences, stories, tools, ways of addressing recurring problems, and many more developed by members.

The original concept of a CoP was based on co-located settings. However, with the growing popularity of the Internet, which makes communication much easier, the concept of virtual communities of practice (VCoP) emerged and was recognized in [31]. A number of papers identified the existence of VCoP and studied different aspects of it [32–35]. Lai et al. [36] provided a thorough literature review and analysis of VCoP. Social Q&A sites are good examples of VCoPs, as they satisfy the three requirements: shared domain of interest, engage in social interactions, and produce shared resources. E.g., in Stack Overflow, first, the shared domain of interest is coding-related questions. Second, users of Stack Overflow interact and learn from each other through the provided Web 2.0 interface. Third, questions and answers remain publicly visible, and are thus shared resources created during problem solving. With the flexibility of social Q&A sites, it is very easy for users to enter a social Q&A site and participate in a VCoP. Likewise, it is also fairly simple to leave the site, thus leaving the VCoP. At the same time, there might exist multiple Q&A sites for the same or similar topics, so users might migrate among different Q&A sites as well. In this paper, we would like to study that given the same or similar topics of knowledge, how people choose which Q&A sites to use, and why people may migrate from one Q&A site to another.

### 2.3 Research questions

Virtual communities of practice (VCoP) exist in *Sec SO*, *Sec SE*, and *Sec Reddit*. The shared domain of interest is cybersecurity-related knowledge, though each site has its own VCoP. The different sizes of the communities, different user configurations, and other factors may lead to site-specific user behaviors among those three communities, especially as the communities differentiate across the three sites. We first study their differential usage patterns. Are questions being answered faster in one community than the others? Are participants in one community more active than participants in the other two communities? Do questions have more answers in one vs. another community?

**RQ1: Are there differences in user behaviors across the three sites?**

Although the general domain of those three VCoP is cybersecurity-related knowledge, each community may have its own idiosyncrasy and focus. For example, the focus of *Sec SO* might be programming-related cybersecurity knowledge, while the focus of the *Sec SE* might be cybersecurity knowledge related to general concepts. A natural question is whether users choose different Q&A sites to post their inquiries on because they evaluate the suitability of one platform or the other for the type of question they have. Next, we study the extent to which this is the case. What types of questions are being asked more in each community? Are there similarities in questions being asked among those communities?

**RQ2: What types of questions are being asked across the three cybersecurity Q&A sites? Are there linguistic differences in the questions asked on one vs the other site?**

As it evolves, a community of practice can go through different stages, including inception, growth, steady state, and decline. Through which of those phases have the three cybersecurity Q&A communities gone? Moreover, when multiple related VCoPs co-exist, they will inevitably end up competing for the same audience during stages of growth, and thus the stages those VCoPs are in may be correlated and even anti-correlated. Do people ever participate in multiple VCoPs, and leave one community for another?

**RQ3: How has the popularity of the three sites, as sources of cybersecurity-related knowledge, been changing over time? Is there a popularity migration from one site to another?**

## 3. Related work

### 3.1 Social Q&A sites

Research on social Q&A sites has been growing since 2009 [13, 37]. Among the numerous social Q&A sites, Stack Exchange [8] in general (and Stack Overflow [9] in particular) has received the most academic attention, measured by the number of published papers per year [38] that use Stack Overflow data. Different aspects of Stack Overflow have been extensively studied, including the prediction of question tags [39, 40], the detection of duplicate questions [41, 42], and the usage of code snippets [43, 44]. Reddit [10] is another very rich source of social community data [45], and many papers [46–49] have studied Reddit as simply a news aggregation and discussion site. Indeed, there are lots of subreddits in Reddit that serve the purpose of news posting and discussion holding. However, some subreddits in Reddit also serve as social Q&A sites, although they have received the attention of fewer, though notable papers [50, 51].

Despite the numerous studies on social Q&A sites and communities, most of the work has focused on a single community at a time. A few papers have studied the different communities of the same topic concurrently, as well as the potential migration among communities. Vasilescu et al. [52] analyzed the differences of user behaviors between two R communities: mailing lists and Stack Exchange (Cross Validated and Stack Overflow), and found evidence of a migration from mailing lists to Stack Exchange due to the latter’s gamified platform. Squire [53] studied the underlying reasons and the effectiveness of 20 open source projects which moved their user support from mailing lists or forums to Stack Overflow. Zagalsky et al. [54] made a comparative study between R community in Stack Overflow and R community in mailing lists regarding types of questions being asked and how knowledge is constructed. Although those related work focused on multiple communities of the same topic concurrently, they focused on communities with totally different structures, such as mailing lists and Stack Overflow. Our paper differs from the existing ones in that we choose three similar structured social Q&A sites as our research domains. We want to study why and how people use three similar structured social Q&A sites to acquire needed cybersecurity knowledge. Moreover, we conduct mixed studies, looking at the communities from different angles, including a linguistic one.

### 3.2 Cybersecurity

Cybersecurity-related challenges affect all areas, including recently burgeoning ones like artificial intelligence (AI) [55], industry 4.0 [56], smart city [57], and internet of things (IoT) [58]. As a result, cybersecurity has attracted the attention of many researchers, and is certainly one of the hottest research topics in computer science. It is impossible to review all those in this space, so we limit ourselves to work that is relevant to knowledge exchange. Because of the increasing number of cyber attacks, how to build a cybersecurity ontology [59–61] has been studied extensively. Another direction of research has been to use Twitter as a rich source of information. For example, Mittal et al. [62] developed a tool to generate alerts for cybersecurity threats and vulnerabilities according to collected relevant tweets. Zong et al. [63] tried to predict the severity of a cybersecurity vulnerability based on the language that is used to describe it in the tweet. Cybersecurity questions contained in social Q&A sites have also been studied. Lippman et al. [64] built classifiers to classify cyber discussions using cybersecurity questions from Stack Overflow as training data. Le et al. [65] proposed a classifier to classify cybersecurity-related questions in Stack Overflow and Security Stack Exchange. Lopez et al. [66, 67] studied how developers use Stack Overflow as the source to solve cybersecurity problems.

## 4. Methods

### 4.1 Data gathering and processing

We integrated data from three different data sources: *Sec SO*, *Sec SE*, and *Sec Reddit*. *Sec SO* means the cybersecurity-related Q&A part of Stack Overflow [9] (questions with a [security] tag), *Sec SE* is the whole Security Stack Exchange site [11], and *Sec Reddit* refers to the three most popular cybersecurity-related Q&A subreddits on Reddit [10] (r/security [24], r/cybersecurity [25] and r/AskNetsec [26]). The data collection methods listed in this section complied with the terms and conditions for the websites from which data was obtained.

All data of every individual site belonging to the Stack Exchange network [8] is publicly available. We used the Stack Exchange API [68] to fetch the data for the Security Stack Exchange site, but there are also other ways to do it, e.g., Data dumps [69] and Stack Exchange Data Explorer [70]. We wrote a python script to automatically call the question API to fetch the real-time information of all questions asked from the beginning of 2011 to the end of 2020. Each question object contains information including the question itself, its corresponding tags, and all its corresponding answers and comments. The creation time and author information of each question/answer/comment are also included. After parsing the API responses, we acquired all the needed information for this paper. We followed a similar procedure for Stack Overflow, but we only kept questions if one of their tags was [security]. We used the same time span (2011-2020) as we did for Security Stack Exchange data. For data from both Stack Overflow and Security Stack Exchange, we exclude closed questions as they comprise a small percentage and do not meet platform guidelines.

We used the well-know Pushshift [71] API [72] to extract data from Reddit. The Pushshift acts as a copy of Reddit objects. Currently, posts and comments are copied into Pushshift’s database at the time they are posted on Reddit (the real-time metadata, such as the number of upvotes, are not available). We wrote a python script to fetch all posts and comments from the three subreddits (r/AskNetsec, r/security, and r/cybersecurity) between 2015 and 2020 inclusive. We chose those three subreddits because they are the most popular subreddits for cybersecurity-related inquiries. The six-years time span was chosen because Pushshift started to collect the data since 2015 [71]. Each post object contains the post id, title, content, author information (username), post type (text or link), and creation time. Each comment object contains the content, author information (username), post id of the post to which this comment belongs, and creation time. After manually inspecting some randomly selected posts from those three subreddits, we found that link posts in those three subreddits relay various cybersecurity-related news, whereas most text posts serve as questions. Because of this, we filtered out all link posts and all comments appearing in link posts. In order to detect non-conforming posts and comments that were later removed by moderators, for each post and comment in our dataset, we fetch its real-time information with the Reddit API [73], using its unique id. Then, we exclude those removed posts and comments that don’t meet the subreddit standard from our dataset. Moreover, we also recover the valid posts, automatically deleted by Reddit spam filters but later restored by moderators.

There exist many bot accounts in Reddit which can post and comment just like humans do. To remove bot posts, we followed a procedure similar to that used in prior work [74], as follows. First, we collected a list of known Reddit bot accounts [75]. Second, we identified all accounts which end with “Bot”, “_bot”, “–bot” or “Modbot” as bot accounts. To reduce false positives, we manually checked those accounts with the user profile and posting history. Finally, we selected very active accounts who have ever posted more than 20 posts or more than 50 comments in those three cybersecurity-related subreddits, and manually verified their identities.

Descriptive statistics of the data we gathered is shown in Table 1. The data from our study is available on: https://zenodo.org/record/5260017. Here, we only show the data statistics of the six overlapping years since the time span of our *Sec Reddit* data is shorter than that of *Sec SO* and *Sec SE*. We restrict the *Sec SO* data and *Sec SE* data to the same time range as *Sec Reddit*’s data for most experiments except RQ3. For *Sec SO* and *Sec SE*, we define “unique participants” as those users who have ever posted questions/answers/comments. For *Sec Reddit*, we define “unique participants” as users who have ever posted posts/comments in *Sec Reddit*.

**Table 1:** Basic statistics for the three data sources.

Sites	Time Span	Posts	Answers	Unique Participants
*Sec SO*	2015-2020	21,260	91,435	39,682
*Sec SE*	2015-2020	35,049	233,330	33,177
*Sec Reddit*	2015-2020	41,207	323,848	61,710

### 4.2 Unifying the different structures of questions among the Q&A sites

The structures of a Reddit text post and a Stack Exchange post are somewhat different. In Reddit, a text post is followed by zero or more “comments”. Each comment can have multiple levels of other nested comments. On the other hand, a Stack Exchange post is followed by zero or more “answers”. Each post, and the answers to it, can have multiple non-nested comments. “Comments” in Reddit are sometimes answers and sometimes are just comments on the post or other comments. To appropriately compare the activity across the three sites, we treat both answers and comments as “answers” for *Sec SO*/*Sec SE* data and treat all comments as “answers” for *Sec Reddit* data, regardless of the nesting level.

### 4.3 Comparing the content of posts across the three Q&A sites

To compare the questions being asked in one site vs. the others, we utilize a qualitative, manual sample examination approach, a quantitative, machine learning/NLP approach, and a quantitative, statistical approach. The goal of the qualitative approach was to identify broad categories (i.e, types) for the questions based on broad concepts in them, and of the quantitative approaches was to determine the similarity between the sites based on linguistic properties of the questions asked on them.

We first sought to identify concrete topical question categories, not only restricted to distinguishing between informational and conversational questions [37]. We chose the six-years time span (2015-2020) that was in common to all the data. From each Q&A site data, we randomly chose 20 posts in the first month of the time span and repeated that every six months. We employed open card sorting [76] to discover the types of questions asked across the three virtual communities. At the beginning, we went through those randomly selected posts from each site and came up with four top-level categories best describing them. This involved several iterations of grouping, ungrouping and re-grouping the categories, until clear divisions were obvious. Then, we manually classified those posts into these four categories to get an overview of the distribution of the types of posts among those three sites.

To determine the semantic similarity between questions on one site and those on another, we first use an NLP approach. BERT [77], which was pre-trained on the BooksCorpus (800M words) [78] and English Wikipedia (2,500M words), is a state-of-the-art language model. We improve BERT’s pre-trained model using an existing approach, Sentence-BERT [79], which uses longer text structures, and involves fine-tuning using different data sets [79]. This approach outperforms other state-of-the-art text embedding methods, including plain BERT, on semantic textual similarity tasks and transfer learning tasks.

We first preprocess all text posts from those three social Q&A sites. Following the practice of [80, 81], we remove the code snippets, URL, and hyperlinks that appear in the texts to avoid the noise. Then for each site, we embed all its text posts into lower dimensional space using Sentence-BERT [79], and calculate the average embedding. This average embedding can be viewed as the semantic embedding of that site. Then, we calculate the cosine difference between each pair of average embeddings to measure the semantic similarity. Although the actual cosine difference value is not meaningful, if the cosine similarity between a pair of websites is larger than the cosine similarity of another pair, then we could say that the first pair is semantically closer than the second pair.

In our second quantitative approach, we use a Venn diagram. For each site, we count the most frequently appearing 100 words, after removing common stop words and lemmatization (removing inflectional endings to revert to base or dictionary form of a word) of all appeared word. Then we draw a 3-circle Venn diagram to visually show the number of overlapping words between the three sites. In general, if a pair of websites has more overlapping words than other pairs, it suggests that this pair is semantically closer than the other pairs of websites.

### 4.4 User survey

We augmented the quantitative experiment with an online user survey to triangulate the quantitative findings. We wanted to understand the real users’ experiences of participating in those three cybersecurity communities and the underlying reasons of the community migration, if they in fact migrated. We asked participants for: if they used each site, how often do they go/participate each Q&A site, the purpose of using each Q&A site, their satisfaction with the answers they get, and their site migration behaviors. The detailed user survey is shown in this link (https://forms.gle/arZWN7ZR6PhLREyk7).

We randomly sampled 1,000 reachable participants (has ever posted/answered/commented a post) from each of the three social Q&A sites. Every Reddit participant is reachable through a private message on Reddit. However, Stack Overflow and Security Stack Exchange do not have the functionality of direct communicating with someone. Instead, we identified reachable participants as those whose emails can be acquired in one of the two ways: (1) directly available on their personal profile page or (2) is available in a corresponding GitHub account when a link to that account is displayed on the user’s personal profile page. Thus, we sent the survey invitations to *Sec Reddit* participants through Reddit’s private message system and sent the survey invitations through email to *Sec SO* and *Sec SE* participants.

When we contacted the participants through email and direct messages, participation consent was informed. We indicated our names, affiliation, the academic purpose of the survey (their responses will be used in a research study), and the voluntary and anonymous feature of the survey. Because of this, our online survey was exempt from the full IRB review as limited surveys of social media participants has been deemed exempt if consent was informed and participation voluntary, which was the case in our study.

## 5. Results

### 5.1 RQ1: Differences in user behavior across the three sites

We first seek to characterize the similarities and differences in question answering, across *Sec SO*, *Sec SE*, and *Sec Reddit*, that are related to user participation. Specifically, we study the fraction of answered questions, the latency to the first answer, the number of answerers/answers per question, and the number of questions asked/answered per unique questioner/answerer. For all experiments in this section, we choose the same six-years time range (2015-2020), available for all three sites. We present both aggregate results and longitudinal results. For all three sites, we exclude answers posted by the questioners themselves in each question.

#### 5.1.1 Answer rate

We start by comparing across the three sites the fraction of questions receiving answers within half year (182 days). Half year is chosen for a fair comparison between questions posted in each year. We include in our counts those deleted questions which were posted in 2015-2020 and deleted after not receiving an answer in 365 days (Stack Overflow [9] and Security Stack Exchange [11] have automatic deletion procedures for questions not receiving any answers in 365 days. After a question is deleted, most of the information are hidden, except the id, creation date, deletion date, and tags). There were 4,322 questions in *Sec SO* and 2,147 questions in *Sec SE* being deleted because of this.

**Fig 1:**
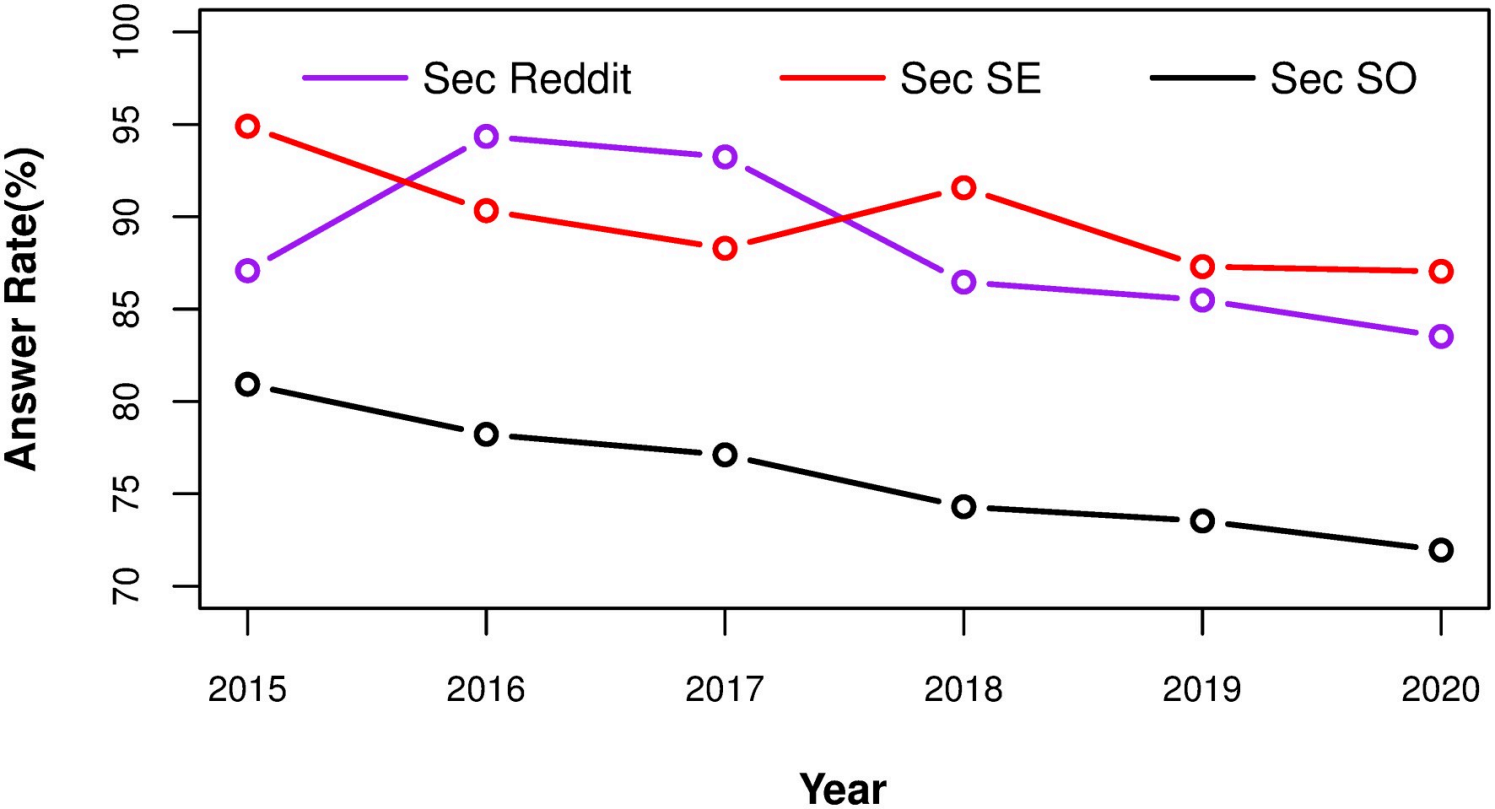
The answer rate of questions being asked each year on *Sec SO*, *Sec SE* and *Sec Reddit*. Black curves are *Sec SO*, Red curves are *Sec SE*, Purple curves are *Sec Reddit*.

The results of questions posted in each individual year and the aggregate numbers are shown in Fig 1 and Table 2, respectively. We see that the answer rate in *Sec SO* is in a steady decreasing trend, while the answer rate in *Sec SE* goes up and down. Meanwhile, for *Sec Reddit*, there is a big jump in answer rate in 2016, and a big percentage fall in 2018.

**Table 2:** Aggregate statistics of the answer rate of questions in each site.

Sites	Total questions	Answered questions	Percentage
*Sec SO*	25,582	19,580	76.54%
*Sec SE*	37,196	33,513	90.10%
*Sec Reddit*	41,207	35,829	86.95%

For the aggregate results, there is a big gap between answer rate in *Sec SO* and answer rates in the other two sites. Only 76.54% questions in *Sec SO* have at least one answer compared to 86.95% and 90.1% for questions in *Sec Reddit* and *Sec SE*, respectively. This is quite surprising because the general user base in Stack Overflow is very large, and most questions with a [security] tag also have other tags. We note that we found 456 questions in *Sec SO*, and 454 in *Sec SE*, that were first answered after they had been posted for more than 182 days, while *Sec Reddit* has no such questions.

#### 5.1.2 Answer latency

Next, we look at the answer latency of questions that receive at least one corresponding answer within 182 days. We define the answer latency as the time interval between the question creation time and the posting time of its first answer.

**Fig 2:**
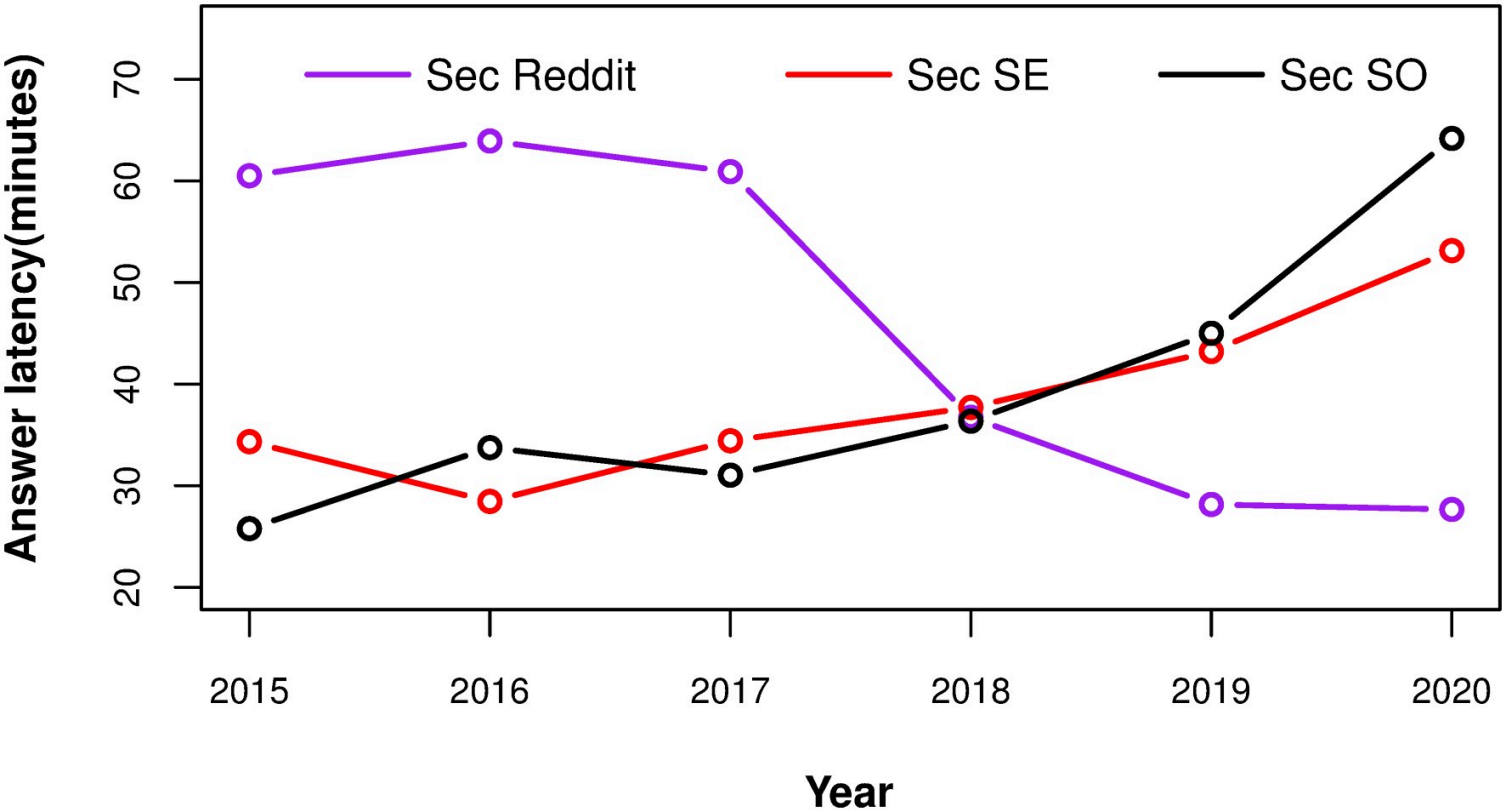
The median answer latency of questions being asked each year on *Sec SO*, *Sec SE* and *Sec Reddit*. Black curves are *Sec SO*, Red curves are *Sec SE*, Purple curves are *Sec Reddit*.

The longitudinal answer latency and the aggregate results are shown in Fig 2 and Table 3, respectively. We show the quartile values for the aggregate data and median value for each individual year because the answer latency of some outlier questions is very large, longer than months. Fig 2 shows clearly that in general, the median answer latency in *Sec SO* and *Sec SE* is growing, especially in 2020, while, at the same time, the median answer latency for Reddit is dropping. In particular, questions on Reddit are answered much faster than before starting 2018.

**Table 3:** Aggregate statistics of the answer latency (in minutes) of questions in each site.

Sites	Q1	Median	Q3
*Sec SO*	7.60	35.73	330.43
*Sec SE*	13.50	36.05	131.83
*Sec Reddit*	14.03	39.93	125.27

The answer latencies of *Sec SE* and *Sec Reddit* are similar, with few minutes differences in Q1, median, and Q3. On the other hand, the statistics of *Sec SO* is markedly different: 25% questions received their first answer within 7.60 minutes while another 25% questions received their first answer after more than 5 hours.

#### 5.1.3 The number of answerers/answers per question

We turn next to the answers and answerers (users who answer the question) of a question. The scope of this analysis is all questions with at least one corresponding answer within 182 days, and we only count answers posted within 182 days.

**Fig 3:**
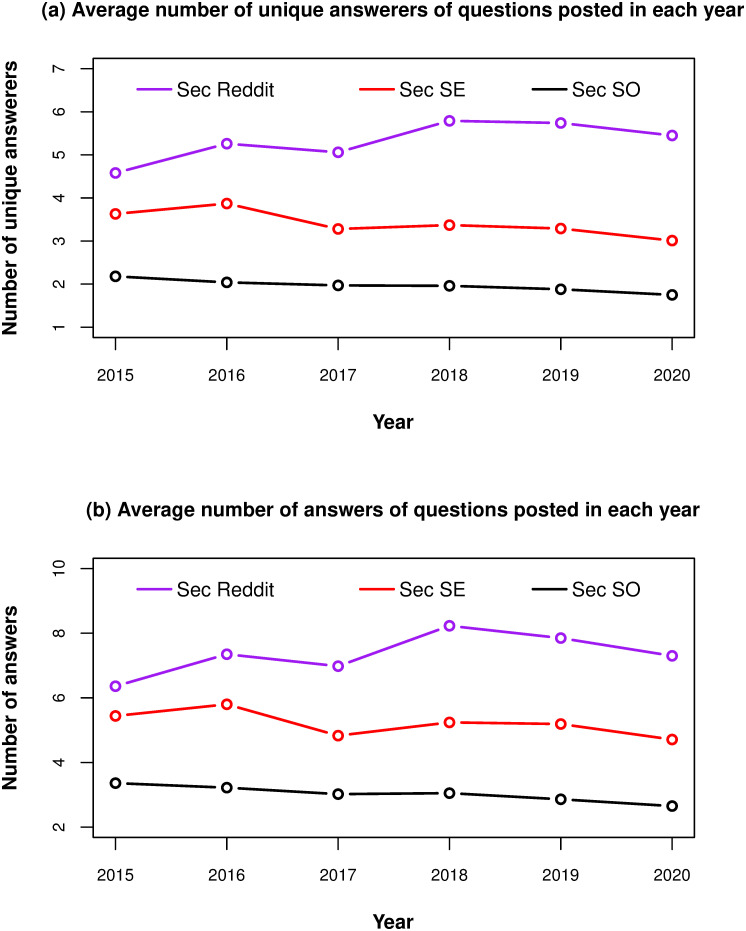
Question answering behavior for questions being asked per year on *Sec SO*, *Sec SE* and *Sec Reddit*. Black curves are *Sec SO*, Red curves are *Sec SE*, Purple curves are *Sec Reddit*.

Fig 3a and 3b show the trends for the average number of answerers and answers, respectively, for questions asked each year across the three sites. The two figures show similar shapes, as expected, because the number of answers in a question equals to the number of answerers multiplied by the number of answers each answerer provides, and the latter number usually does not vary much. In general, the curves for *Sec SO* and *Sec SE* are relatively stable over time, especially the former. The only exception is that there is a big drop in 2017 for *Sec SE*. The variation in the curve for *Sec Reddit* is larger than it for *Sec SO* and *Sec SE*, and there are two big boosts appearing in 2016 and 2018.

From the aggregate statistics, in Table 4, we see that the mean value and the median value of the number of “unique” answerers in a question for *Sec Reddit* are 5.29 and 3, higher than those for questions asked in *Sec SE*, and much higher than those of *Sec SO*. The number of answers given by those “unique” answerers shows a similar pattern, with *Sec Reddit* numbers higher than *Sec SE* and *Sec SO*.

**Table 4:** Aggregate statistics of the question answering behavior.

Sites	Q1	Median	Q3	Mean	STD
**Number of “unique” Answerers in a question**					
*Sec SO*	1	2	2	1.99	1.35
*Sec SE*	1	2	4	3.46	4.57
*Sec Reddit*	2	3	6	5.29	7.08
**Number of answers in a question**					
*Sec SO*	1	2	4	3.07	2.72
*Sec SE*	2	3	6	5.25	7.58
*Sec Reddit*	2	4	8	7.29	11.61

#### 5.1.4 Participation behavior

The number of unique questioners (users who post questions) and unique answerers (users who answer questions) is shown in Table 5. It is interesting that for *Sec SE*, there are only 15,171 unique answerers, much less than the 21,656 unique questioners. For the other two sites, the number of answerers is higher than the number of questioners, especially in *Sec Reddit*, and by a large margin. The results for *Sec SO* and *Sec Reddit* are somewhat expected because there are 1.99 answerers for a *Sec SO* question on average, and there are 5.29 answerers for a *Sec Reddit* question. The surprisingly low ratio of the number of unique answerers to the number of unique questioners in *Sec SE* suggests that on average, each answerer in *Sec SE* answers many more questions than answerers in *Sec SO* and *Sec Reddit*.

**Table 5:** Aggregate statistics of the number of unique questioner/answerer in each site.

Sites	Total unique questioner	Total unique answerer
*Sec SO*	18,882	21,670
*Sec SE*	21,656	15,171
*Sec Reddit*	26,479	41,682

**Fig 4:**
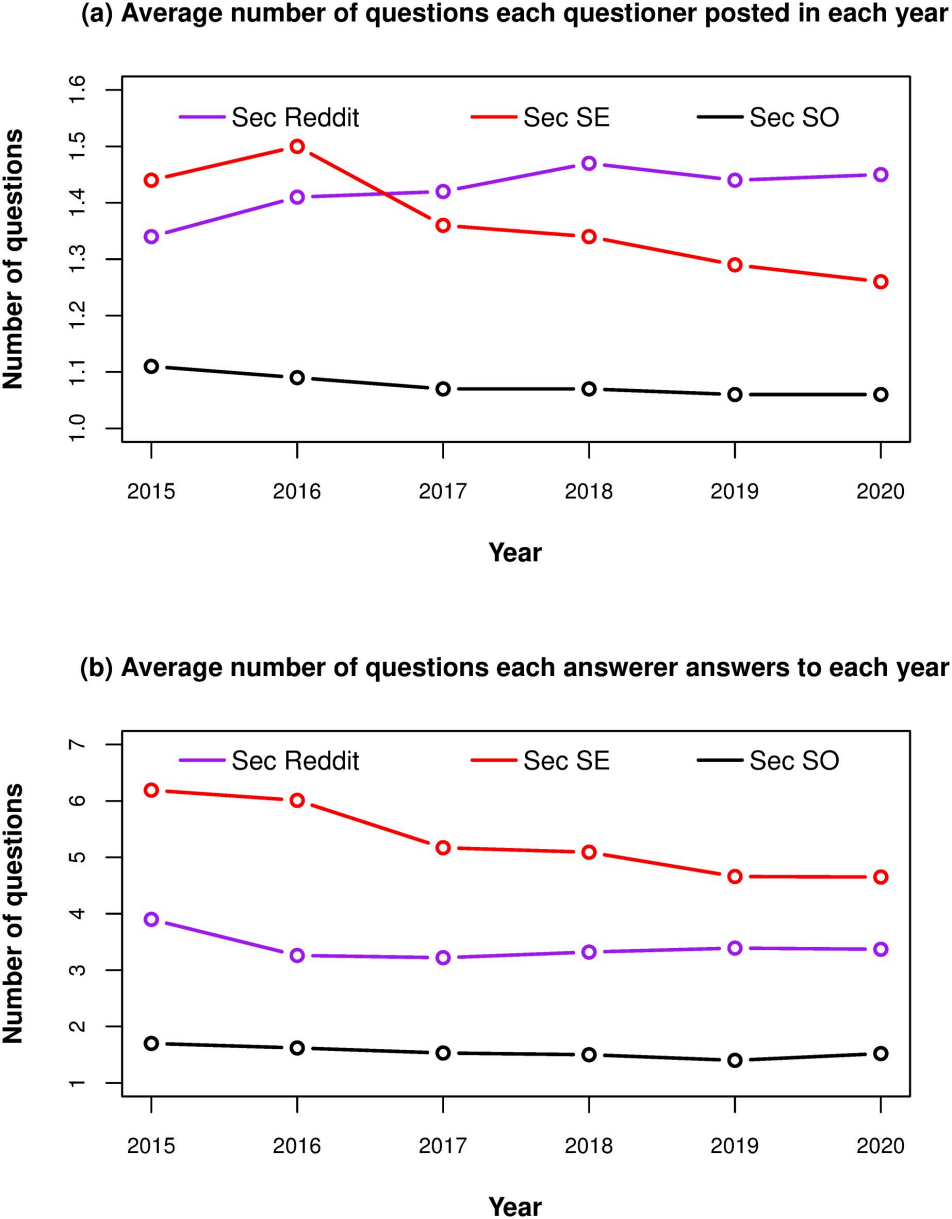
Participation behavior of contributors per year on *Sec SO*, *Sec SE* and *Sec Reddit*. Black curves are *Sec SO*, Red curves are *Sec SE*, Purple curves are *Sec Reddit*.

The participation behavior per year is shown in Fig 4. Questioners in *Sec SO* show a smooth decreasing trend, while the corresponding *Sec SE* curve increases in 2016 and starts decreasing after that. *Sec Reddit* shows a generally increasing trend. Answerer numbers decrease in *Sec SO* and *Sec SE* starting in 2015, and to a lesser extent in *Sec SO*. The *Sec Reddit* curve drops drastically in 2016, and then starts climbing up at a slower rate.

The aggregate number of questions asked per questioner and the number of questions answered per answerer for the whole five-years time periods are shown in Table 6.

**Table 6:** Aggregate statistics of the contributor behavior.

Sites	Q1	Median	Q3	Mean	STD
**Questions asked per questioner**					
*Sec SO*	1	1	1	1.13	0.53
*Sec SE*	1	1	1	1.62	2.43
*Sec Reddit*	1	1	1	1.53	2.34
**Questions answered per answerer**					
*Sec SO*	1	1	1	1.91	7.20
*Sec SE*	1	1	3	8.08	63.59
*Sec Reddit*	1	1	3	4.35	15.93

We first look at the behavior of questioners. The first quartile, median, and third quartile for questioners in all those three sites are 1, so we use the unpaired t-test to assess whether the difference in the means is significant. The low P-values (< 0.0001) for each pair (Table 7) show that the means are in fact very likely different, implying different behavior.

**Table 7:** Two tailed P-value for the statistics of the questions asked per questioner.

Sites	Sites	Two tailed P-value
*Sec SO*	*Sec SE*	<0.0001
*Sec SO*	*Sec Reddit*	<0.0001
*Sec SE*	*Sec Reddit*	<0.0001

Table 6 shows that more than 75% questioners in each site only ask one question. For *Sec SO*, the 1.13 mean value and the 0.53 standard deviation value show that most questioners only ask one question with few exceptions. The values for *Sec SE* and *Sec Reddit* are similar, with relatively large standard deviation values. This implies that there exists a small number of questioners in *Sec SE* and *Sec Reddit* asking more questions.

Then we look at the number of unique questions each answerer answers to in Table 6. The result is very telling. For *Sec SE*, given the other values, the mean of 8.08 and the standard deviation of 63.59 are surprisingly large. This suggests the existence of a small portion of answerers, each answering a large number of questions.

We analyze the above distributions at a finer level by looking at the distribution of answerers answering different numbers of questions. The six intervals are 0-5, 6-10, 11-50, 51-100, 101-500, and >500. The result is displayed in Table 8. For *Sec SO*, 96.45% answerers answer 0-5 questions, with few percent of answerers answering more questions. For *Sec Reddit*, 86.45% answerers answer 0-5 questions, 6.51% answerers answer 6-10 questions, and 6.05% answerers answer 11-50 questions. There are also 0.99% (414) very active answerers answering more than 50 questions. Regarding *Sec SE*, the percentages are close to those of *Sec Reddit* for answerers answering 0-5, 6-10, and 11-50 questions, but the percentage of very active answerers answering more than 50 questions in *Sec SE* is more than double the percentage for *Sec Reddit*. In particular, there are 2.50% (379) answerers answering more than 50 questions each in *Sec SE*. Since we didn’t include deleted low-quality answers in our dataset, the above percentage could suggest the existence of many active and knowledgeable answerers who supply many good answers in *Sec SE*, and thus a higher level of expertise in cybersecurity.

**Table 8:** Aggregate statistics of distributions across the three sites of answerers answering different number of unique questions.

Sites	0-5	6-10	11-50	51-100	101-500	>500
*Sec SO*	96.45%	1.99%	1.33%	0.14%	0.08%	0.00%
*Sec SE*	84.87%	6.05%	6.58%	1.23%	1.09%	0.18%
*Sec Reddit*	86.45%	6.51%	6.05%	0.63%	0.34%	0.02%

**RQ1: Many cybersecurity questions in *Sec SO* are answered very quickly but many others are answered very slowly or left unanswered; Questions asked in *Sec Reddit* have more answerers and answers than those in the other two sites; The percentage of more active and knowledgeable answerers on *Sec SE* is higher than that of *Sec Reddit*, and both are much higher than that of *Sec SO*. However, *Sec Reddit* activity is increasing over time while *Sec SO* and *Sec SE* are decreasing, especially the latter**.

### 5.2 RQ2: Types of questions asked and linguistic differences

#### 5.2.1 Types of questions being asked across the three Q&A sites

Using open card sorting on samples of the data from each site, as described in the Materials and methods section, we found that there are 4 main types of questions on the three sites:

- Type1: Look for solutions about cybersecurity-related programming questions.
- Type2: Look for solutions about cybersecurity-related general questions.
- Type3: Look for advice of career/resume/interview regarding cybersecurity field.
- Type4: Look for recommendations or user experiences of cybersecurity-related products/software/tutorials/courses.

The most popular two types are informational questions [37] where people are looking for solutions about cybersecurity-related problems. The difference is if the question is programming-related or not. An example of Type 1 question [82, 83] is when one posts a snippet of code and asks whether his code is free from the directory traversal attack. In contrast, a Type 2 question [84, 85] might ask why one would want a timeout on a server and what is the security benefit of it. Thus, this is a general concept question.

The next two categories of questions are conversational questions [37] that are less technical and more subjective as there might be no ground truth or correct answers to those categories of questions. For instance, a Type 3 question [86, 87] might ask about entry-level jobs in the netsec/infosec field and how to get started in the field. Lastly, a Type 4 question [88, 89], might ask for learning recommendations for Enterprise PKI design and management, especially some books or web resources. This question is still cybersecurity-related, but related to educational or training resources.

The manual classification result of those randomly selected 240 questions for each platform is displayed in Table 9. The distributions of the types of questions asked across the Q&A sites are very different. For *Sec SO*, the first two more objective types of questions contribute to 94.58% of total questions, and 45% of the total questions are programming-related. Similarly, the first two types of questions in *Sec SE* occupy 91.67% of all questions, with only 17.5% of the total questions are programming-related. The statistics for *Sec Reddit* is very different. The first two types comprise 46.67% of the total questions, though only 1.67% for Type 1. There are 19.58% of questions on *Sec Reddit* that are career-related inquiries, while the percentage is 0% and 0.42% for *Sec SO* and *Sec SE*. The fourth type of questions in *Sec Reddit* contributes to 33.75% of the total questions which is much higher than that of *Sec SO* and *Sec SE*. The underlying reasons might be that *Sec SO* and *Sec SE* have more strict requirements about what should be asked and should not be asked on the sites, whereas *Sec Reddit* does not have those restrictions. The very low percentage of Type 3 questions on *Sec SO* and *Sec SE* may be due to the fact that career-related inquiries are mostly opinion-based, and this is one of the five reasons for questions being closed in Stack Overflow and Security Stack Exchange. The situation for Type 4 questions is similar, and we found some closed Type 4 questions in *Sec SO* and *Sec SE* during our manual inspection. “Opinion-based” and “off-topic” are two common reasons for Type 4 questions being closed.

**Table 9:** The manual classification result of the distributions of the four types of questions in each Q&A site.

Sites	Type1	Type2	Type3	Type4
*Sec SO*	45.00%	49.58%	0.00%	5.42%
*Sec SE*	17.50%	74.17%	0.42%	7.92%
*Sec Reddit*	1.67%	45.00%	19.58%	33.75%

#### 5.2.2 Semantic similarity across the three Q&A sites

The results shown in Table 10 are cosine similarities of average Sentence-BERT [79] sentence embeddings between questions posted in different years of the same site. We see that there is a high semantic similarity between questions posted in different years on each site. This suggests that for each site, the questions asked over time do not vary much semantically.

**Table 10:** Cosine similarity(in-domain) of average SentBert embeddings between questions asked in different years of each site.

**Sites & Year**	**Sites & Year**	**Cos Sim(%)**
*Sec SO* 2015	*Sec SO* 2018	99.93
*Sec SO* 2018	*Sec SO* 2020	99.94
*Sec SO* 2015	*Sec SO* 2020	99.85
**Sites & Year**	**Sites & Year**	**Cos Sim(%)**
*Sec SE* 2015	*Sec SE* 2018	99.87
*Sec SE* 2018	*Sec SE* 2020	99.95
*Sec SE* 2015	*Sec SE* 2020	99.85
**Sites & Year**	**Sites & Year**	**Cos Sim(%)**
*Sec Reddit* 2015	*Sec Reddit* 2018	99.74
*Sec Reddit* 2018	*Sec Reddit* 2020	99.77
*Sec Reddit* 2015	*Sec Reddit* 2020	99.48

Then, we compare the cosine similarities of embeddings of questions posted in the same year between each pair of websites. Table 11 shows that *Sec SO* and *Sec SE* are more semantically similar than any other two pairs of the three sites. The result is not surprising as the first two types (see above) of similar questions contribute to 94.58% and 91.67% questions on *Sec SO* and *Sec SE*, and we already removed any code in the pre-processing step of this experiment. Another interesting finding is that questions on *Sec Reddit* are semantically closer to *Sec SE* than to *Sec SO*. The potential reason behind this is that only 1.67% of the questions in *Sec Reddit* are programming-related, whereas a large fraction of questions on *Sec SO* are programming-related. We also observe that *Sec SO* and *Sec SE* are becoming semantically closer to each other over time, while *Sec Reddit* is becoming more distant from both.

**Table 11:** Cosine similarity(across-domain) of average SentBert embeddings between questions asked in same year of different sites.

**Sites & Year**	**Sites & Year**	**Cos Sim(%)**
*Sec SO* 2015	*Sec SE* 2015	97.52
*Sec SO* 2018	*Sec SE* 2018	98.03
*Sec SO* 2020	*Sec SE* 2020	98.05
**Sites & Year**	**Sites & Year**	**Cos Sim(%)**
*Sec SE* 2015	*Sec Reddit* 2015	96.15
*Sec SE* 2018	*Sec Reddit* 2018	95.32
*Sec SE* 2020	*Sec Reddit* 2020	93.50
**Sites & Year**	**Sites & Year**	**Cos Sim(%)**
*Sec SO* 2015	*Sec Reddit* 2015	94.61
*Sec SO* 2018	*Sec Reddit* 2018	93.63
*Sec SO* 2020	*Sec Reddit* 2020	92.02

**Fig 5:**
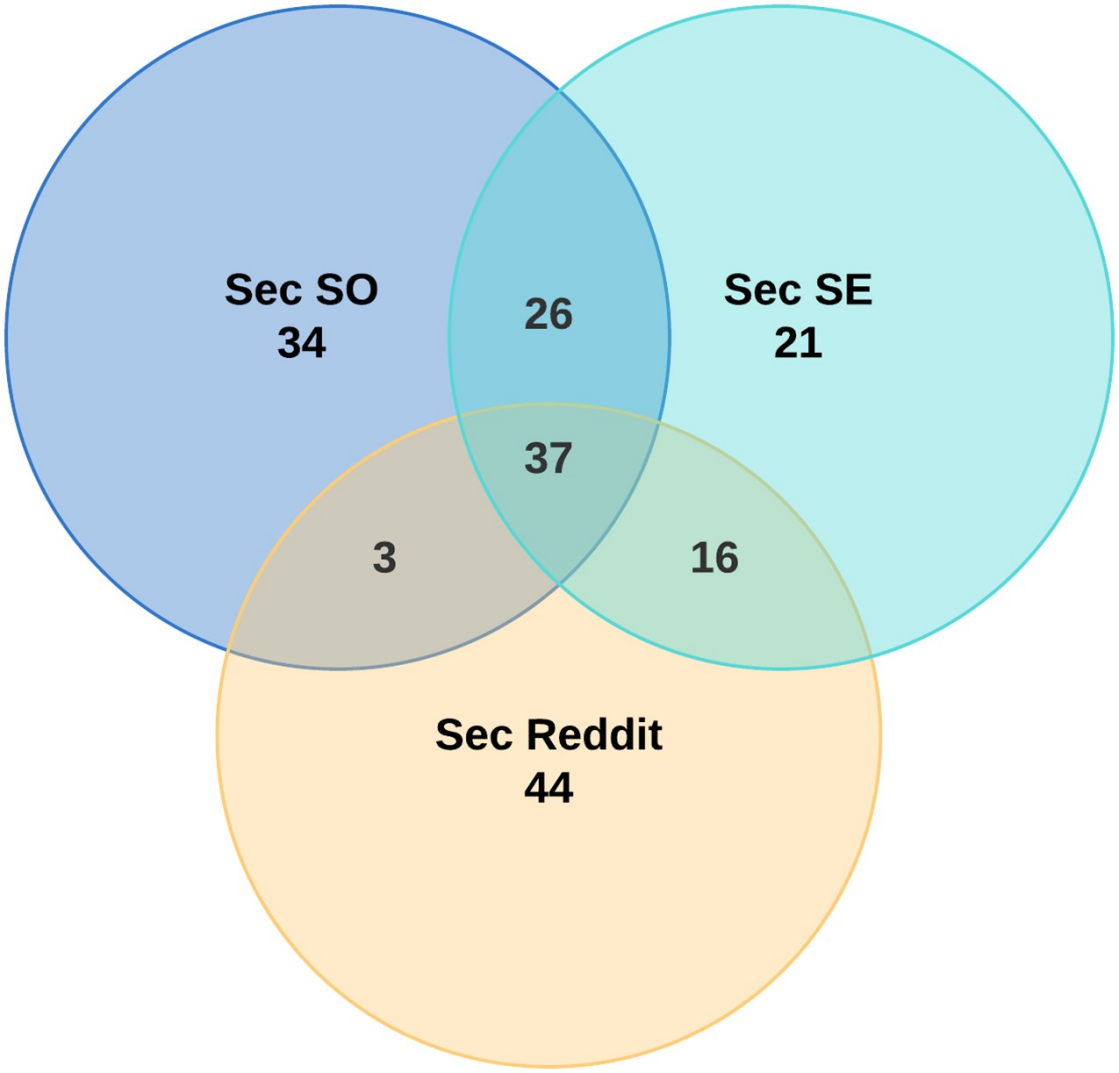
The Venn diagram of the most frequent 100 words of each site.

Fig 5 presents a 3-way Venn diagram of the most frequent 100 words of each site. Besides the 37 overlapping words among all those three sites, *Sec SO* and *Sec SE* have 26 additional overlapping frequent words, more than *Sec SE* and *Sec Reddit* (only 16). *Sec SO* and *Sec Reddit* have only 3 additional overlapping frequent words, the least. This is consistent with the above findings that *Sec SO* and *Sec SE* are most semantically similar.

**RQ2: Most questions on *Sec SO* and *Sec SE* appear to be technical, with those on *Sec SO* focusing more on programming. *Sec Reddit* includes both technical and more subjective or opinion-based questions. Questions on *Sec SO* and *Sec SE* are more semantically similar to each other, than questions on *Sec Reddit*, and growing closer over time. *Sec Reddit* questions are growing more semantically distant from the others**.

### 5.3 RQ3: Popularity change over time and user migration

First, we use the number of questions asked, the number of answers given, the number of unique questioners, and the number of unique answerers as indicators for the popularity change of the three sites over time, in monthly intervals.

**Fig 6:**
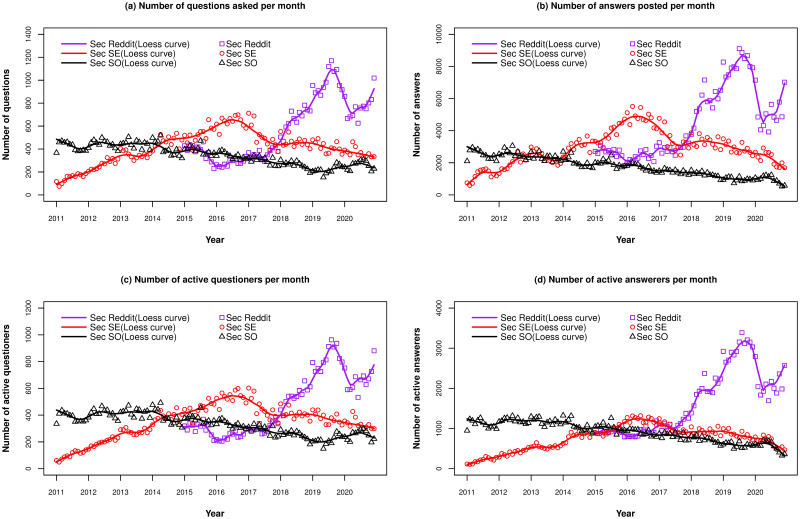
The popularity trends per month for *Sec SO*, *Sec SE*, and *Sec Reddit*. The trend curves are Loess curve with 0.1 spin. Black curves are *Sec SO*, Red curves are *Sec SE*, Purple curves are *Sec Reddit*.

Fig 6(a)–6(d) show the monthly trends for each site, for the four popularity measures. Each shows qualitatively the same story: the popularity of *Sec SO* is in a generally decreasing trend from 2011 on, though the curve is not very steep. One potential reason is that with the advent of other social Q&A sites hosting cybersecurity-related questions, especially *Sec SE*, some questioners may turn to ask their cybersecurity questions on social Q&A sites other than *Sec SO*. For *Sec SE*, the popularity increases from 2011 to 2016 and reaches its peak during the second half of 2016. This is what we would expect as the *Sec SE* was created in the second half of 2010. As the site’s popularity increased throughout the years, it likely attracted many questioners from *Sec SO*. However, since 2017, its popularity has been decreasing. On the contrary, the number of questions being posted on *Sec Reddit* does not vary much from 2015 to the first half of 2017, but after that, the number has been growing super-linearly until the end of 2019. In the first half of 2020, there is a big drop in the popularity of *Sec Reddit*. One potential reason might be that r/security [24], one of the three subreddits that we focus on, was shut down at that time and is still under construction up to now. Although it mentions that people should instead visit r/cybersecurity [25], users of r/security may not migrate that quickly or might migrate to other subreddits. In the second half of 2020, the popularity starts climbing again as possibly most users of r/security migrated to the r/cybersecurity community. Some reasons for the popularity change will be revealed in the next section on the survey results.

Next, we study migrations of users between *Sec SO* and *Sec SE*, based on their activity in both. We can do that for *Sec SO* and *Sec SE* because they both belong to the Stack Exchange network [8]. If a user registers on both Stack Overflow [9] and Security Stack Exchange [11], his site-wise unique identifiers will both link to a globally unique Stack Exchange network identifier. Of the 215,040 registered users from Security Stack Exchange, 168,499 (78.36%) also have Stack Overflow accounts. Of those 168,499 overlapping users, 92.07% (155,143) created their Stack Overflow account before they created their Security Stack Exchange account.

**Fig 7:**
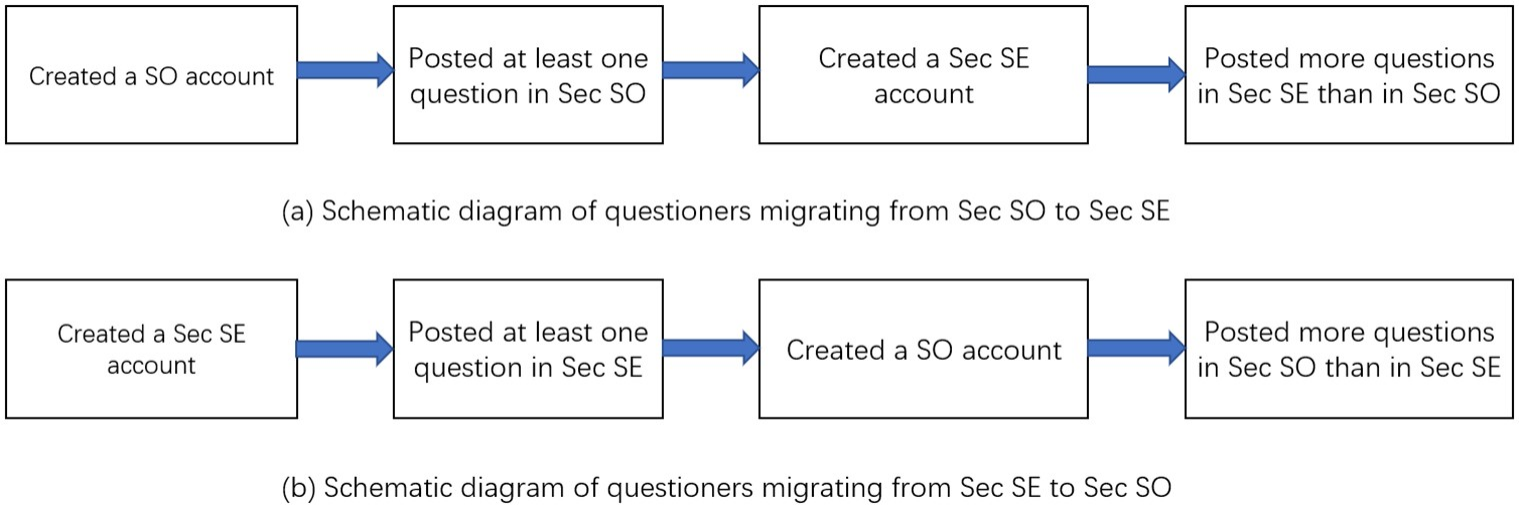
Schematic diagram of migrating behavior.

We limit our scope to only questioners and answerers since we only detect activity when a user posts something (browser views are not publicly available from any of these sites). We define a *migration* from *Sec SO* to *Sec SE* to be when a questioner who has created his SO account before his *Sec SE* account, posts at least one question in *Sec SO* before he created his *Sec SE* account, and posts more questions in *Sec SE* than in *Sec SO* after the creation of his *Sec SE* account (see Fig 7(a)). The definition of migration from *Sec SE* to *Sec SO* is similar (see Fig 7(b)). The migration for an answerer is defined the same as for a questioner, except now we count the number of questions answered.

With that, we counted 1,677 questioners who migrated from *Sec SO* to *Sec SE*, and only 26 cases for the reverse migration, as shown in Table 12. Likewise, 2,041 answerers migrated from *Sec SO* to *Sec SE*, and only 14 for the reverse.

**Table 12:** The statistics of contributor migration behaviors.

Migrate from	Migrate to	User type	Number
*Sec SO*	*Sec SE*	Questioner	1,677
*Sec SE*	*Sec SO*	Questioner	26
*Sec SO*	*Sec SE*	Answerer	2,041
*Sec SE*	*Sec SO*	Answerer	14

**RQ3: *Sec SO* has been losing its popularity for cybersecurity questions at a constant rate since 2011. *Sec SE* had been gaining in popularity from 2011 to 2016, and then has started losing it since 2017. *Sec Reddit* has been flourishing rapidly since the second half of 2017, and in 2020, its popularity dropped down and bounced back. Lastly, we found that many contributors have migrated from *Sec SO* to *Sec SE***.

### 5.4 Triangulation survey

We triangulate the previous quantitative findings with the results from a user survey described in the Methods section. We received 45 valid responses to our survey (46 total responses, but one respondent participated twice). 13 responses were from *Sec Reddit* contributors, 19 from *Sec SE* contributors, and 13 from *Sec SO* contributors. Of the 45, 19 (42.22%) stated they have used all three sites and maybe others, 16 (35.56%) used two of the three, 6 (13.33%) used only one, and 4 gave no answer.

To our question “If you have ever switched from most frequently using one to most frequently using another of these three websites for “cybersecurity-related” questions and answers, which one did you switch from and to?”, we offered six answers corresponding to all pairwise potential migration between the three websites. The result is shown in Table 13. Of 25 participants who reported their migration behaviors, 19 reported leaving *Sec SO*, with 16 going to *Sec SE* as the destination. This validates the result of our quantitative experiment. 11 of those 16 people provided reasons, the main being that *Sec SE* is better in terms of findings things or the quality of answers.

**Table 13:** The migration behaviors and reasons reported by survey participants.

Source	Migration	Answers
*Sec SO*	*Sec SO* to *Sec SE*	“Quantity and quality of answers”
	*Sec SO* to *Sec SE*	“security.stackexchange.com did not exist in its full form in the past”
	*Sec SO* to *Sec SE*	“security.stackexchange.com is more specifically about security than stackoverflow”
	*Sec SO* to *Sec SE*	“SO is code focused but sec. stackexch. is concept focused”
	*Sec SO* to *Sec SE*	N/A
	*Sec SO* to *Sec SE*	N/A
	*Sec SO* to *Sec SE*	N/A
*Sec SE*	*Sec SO* to *Sec SE*	“I found better answers on the other site.”
	*Sec SO* to *Sec SE*	“security.stackexchange.com seems to better reach the experts even though its user base is a lot smaller than stackoverflow.com. For simple questions stackoverflow.com seems to work better even if the question was security related because of its larger user base.”
	*Sec SO* to *Sec SE*	“The search engine replies just happened to point to security.stackexchange.com more often.”
	*Sec SO* to *Sec SE*	N/A
	*Sec SO* to *Sec SE*	N/A
	*Sec SE* to *Sec SO*	N/A
	*Sec SO* to *Sec Reddit*	“The format of “top answer” often promotes incomplete answers; and users get upset about someone else providing additional answers, even if they are better.”
	*Sec Reddit* to *Sec SO*	“Generally, my experience with Reddit is that it’s a lot of talk but not much by way of answers. The place to find concrete, understandable answers is almost always Stack Overflow.”
*Sec Reddit*	*Sec SO* to *Sec SE*	“Better reply”
	*Sec SO* to *Sec SE*	“Focused discussions”
	*Sec SO* to *Sec SE*	“Just better for it in terms of finding things”
	*Sec SO* to *Sec SE*	“I couldn’t exactly find the right answer and needed more background”
	*Sec SO* to *Sec Reddit*	“More welcoming community. More informative answers. More reliable response rate.”
	*Sec SO* to *Sec Reddit*	“Solving a security issue”
	*Sec Reddit* to *Sec SE*	N/A
	*Sec Reddit* to *Sec SE*	N/A
	*Sec Reddit* to *Sec SO*	N/A
	*Sec SE* to *Sec Reddit*	“Lack of replies”

This reason is supported by the answers of other two survey questions. The first question is “If you use that site for seeking answers, how often do you find the replies to the existing questions can fulfill your need?” (Likert scale, 1—I seldom find what I need, 5—I always find what I need) The second question is “If you have ever posted questions on that site, how satisfied or dissatisfied are you with the answers/advice/recommendations you get?” (Likert scale, 1—Very dissatisfied, 5—Very satisfied). We analyzed the answers to those two questions, and came up with average Likert scores for each. (Higher is better according to our question design.) The score of those two survey questions for *Sec SO* is 3.34 and 3.5, whereas the score for *Sec SE* is 4 and 3.79. The score for *Sec SE* exceeds that of *Sec SO* which supports the main potential migration reason.

Another main reason supported by some participants is that *Sec SE* is designed specifically for cybersecurity-related questions and answers, and is better to reach the experts. This reason matches our earlier quantitative findings and corresponds with our theory of the formation of community of practice.

**Fig 8:**
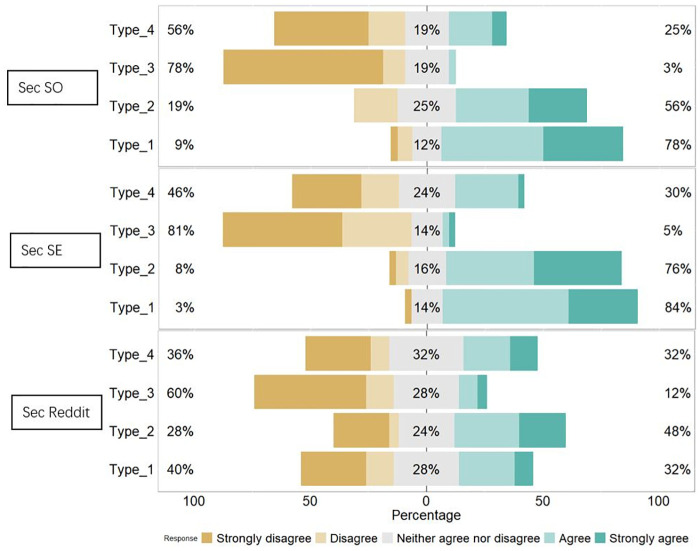
The agreement of participants using each site for four types of questions and their corresponding answers.

Our next empirical finding is about how people use each of the Q&A sites for different types of questions and their corresponding answers. In the survey, we asked participants the reason for using each site if they have ever used that site before. We listed the description of each type of question and the answer is in Likert scale. (1-strongly disagree to 5-strongly agree). The summarized responses for *Sec SO*, *Sec SE*, and *Sec Reddit* are shown in Fig 8.

Generally speaking, the responses match with our manual classification results. As we can see, for *Sec SE* users, 84% of them agree that Type 1 questions are their purpose and 76% of them agree on that Type 2 questions are their purpose. For *Sec SO* users, the levels of agreement are 78% and 56% regarding Type 1 and Type 2 questions. Indeed, most questions in *Sec SO* and *Sec SE* are Type 1 and Type 2 questions. The bigger difference between Type 1 and Type 2 questions in *Sec SO* than in *Sec SE* corresponds to the higher percentage of the Type 1 question in *Sec SO* by our manual classification result. The levels of agreement are fairly low regarding Type 4 questions, and even less so (<5%) for Type 3 questions among users from *Sec SO* and *Sec SE*. This is not surprising as the percentage of Type 4 questions is less than 8% in those two sites, and less than 0.5% of questions in those two sites are Type 3. For the responses from *Sec Reddit* users, we can clearly see that they have much higher disagreements on what types of questions they use *Sec Reddit* for than users from *Sec SO* and *Sec SE*. Relatively more users use *Sec Reddit* for Type 2 questions, while Type 4 questions match with the larger percentages of Type 2 and Type 4 questions in *Sec Reddit*. The only difference here is that although Type 3 questions in *Sec Reddit* occupy 19.58% of the questions, the agreement on Type 3 questions for *Sec Reddit* users is fairly low. One potential reason is that career-related inquiries are very individual, and the randomly chosen participants might not have the need to ask career-related inquiries.

## 6. Discussion

### 6.1 Implications

In the previous sections, we provided evidence for the usage and linguistic differentiation of the three sites, suggesting they may be used by different communities. Here we discuss the underlying reasons for the differentiation.

Although the interface, functionality, and the gamification mechanisms employed in SO and Security SE are exactly the same, our experiments showed significant differences in user/contributor behaviors between *Sec SO* and *Sec SE*. One reason for that might be the different focus of the two communities. The SO community has a wider focus, on general programming-related, technical questions. *Sec SO* is a very small portion of SO and the [security] tag is overshadowed by numerous more popular tags. Table 4 shows that the average number of answerers and answers for questions in *Sec SO* are smaller than those in *Sec SE*. On the other hand, the focus in *Sec SE* is narrower, limited mostly to cybersecurity-related technical questions. Our result (Tables 6 and 8) seems consistent with that: the community with a more specific focus on cybersecurity tends to attract more cybersecurity experts who can answer lots of questions, whereas most answerers in *Sec SO* only answer one question each. In fact, *Sec SE* has a 13.56% advantage over *Sec SO* in answer rates (Table 2), and most questions are answered within two hours (Table 3). Although some portions of relatively “hard” questions in *Sec SO* are left unanswered or answered after several hours, there are also some others being answered more quickly than in *Sec SE* (Table 3). A potential explanation for this is given the very broad focus, the user base in Stack Overflow is large, so relatively “easy” cybersecurity questions are answered quickly.

*Sec SE* and *Sec Reddit* both focus on cybersecurity, and have similar answer latencies (Table 3) and answer rates (Table 2) indeed. However, *Sec SE* and *Sec Reddit* differ a lot in the average number of answerers/answers per question (Table 4). We propose that two different design choices may be at the root of this differentiation. The first is in the gamification system. Each SE site employs the same reputation system, where a user gets reputation points as a measurement of the community trust in them. Reputation increases by providing quality questions and answers, so it could be an important sign of ability and expertise. Reddit also has a gamification system, the karma points, they do not have the same meaning since providing clickable, funny, or interesting content can also earn karma points. The importance of the reputation system in *Sec SO* and *Sec SE* urges users to compete with each other by providing faster and better answers. Once an answer is accepted by the questioner, other users might not want to answer the question anymore as their answers have small chances of being selected as the accepted answer.

The second reason might be that *Sec Reddit* does not place any requirements on what should be asked, whereas *Sec SO* and *Sec SE* are designed for only certain types of questions. Our classification (Table 9) shows that roughly half of *Sec Reddit* questions are of the subjective and discussion types, compared to 5.42% and 8.34% in *Sec SO* and *Sec SE*, respectively for the same. Those types of questions tend to generate more answers than objective ones.

Our work can also facilitate the match between people’s cybersecurity, and even social, needs and the available social Q&A sites. According to our classification result (Table 9), conversational questions, especially those about career or resources recommendations, should be asked on *Sec Reddit*, while informational questions are more in line with *Sec SO* and *Sec SE*, with coding-related questions more suitable in *Sec SO* and concepts-related questions more fit in *Sec SE*. Informational questions could be asked on *Sec SO* for a faster reply (Table 3) due to Stack Overflow’s large user base, while more technical questions could be asked on *Sec SE* as there are more active and knowledgeable answerers there (Table 8).

### 6.2 Limitations

The combined quantitative analysis and the user survey have allowed us to finely analyze the empirical data and derive strong conclusions. We do however acknowledge a number of threats to our approaches. The biggest potential threat to validity resides in the process of data gathering and processing. First, we extracted data from Stack Overflow [9] and Security Stack Exchange [11] using the provided API. However, most of the information about deleted questions and answers are not available to the public. Second, we applied reasonable filtering of Reddit bot accounts and their associated posts and comments, but we do not know whether all bot accounts have been identified or not unless we examined each account one by one. Third, there might be throwaway accounts [90] used in Reddit [10]. However, according to [90], throwaway accounts are used mainly for disclosing personal or controversial information, which is not the common case in cybersecurity-related Q&A. Finally, selection bias might exist in choosing the scope of the cybersecurity community. There could potentially be cybersecurity-related Q&As in less popular subreddits and perhaps some cybersecurity-related questions in Stack Overflow without a [security] tag. However, we think that the majority of the cybersecurity-related questions on those two sites are included in our study.

Threats in user survey are that there may also exist selection bias in selecting the survey participants and the survey responses might come from self-selecting participants, although we believe this threat is lower since we only use the survey results to triangulate our quantitative findings, and not on their own.

Another potential limitation is whether our results can be generalized to other Q&A sites and to other domains, e.g., to the domain of linux-related Q&A among Unix&Linux Stack Exchange [91], Stack Overflow (questions with [linux] tag), and Reddit subreddit (r/linux [92]). This is one future research direction.

Finally, due to the anonymity inherent to the site, we could not trace users migrating between *Sec Reddit* and the other two sites in our study. Thus, we can only conclude that the popularity of *Sec Reddit* is booming since 2017, and are not sure where those newcomers come from. Our survey also does not explain the flourishing of *Sec Reddit* and its underlying reasons because in it there was the same number of respondents migrating to Reddit and migrating out of it. This is another direction for further research.

## 7. Conclusion

In this paper, we conducted a mixed method empirical study of the user behaviors, question types and content, and popularity trends over time for the cybersecurity communities in three popular question and answer sites: Stack Overflow, Security Stack Exchange, and Reddit. The novelty of our approach was in considering multiple sites within the communities of practice framework, thus having their identifiable characteristics. We found that the cybersecurity-related questions asked on the three sites are different both in type and content, with Stack Overflow and Security Stack Exchange having more technical, while Reddit having more subjective and personal interest questions. Moreover, we found that there appears to have been an interesting popularity trade between these sites over time, accompanied by a migration of people between them, strongly suggesting that Stack Overflow has fallen out of favor within the cybersecurity community, Security Stack Exchange has cemented itself as the place to be for expedient and high-quality answers to cybersecurity-related questions, and Reddit is addressing the more subjective, discussion type needs of the lay community. This is consistent with the CoP theory of commonality and differentiation over time. Future directions along these lines are (1) to understand more fundamentally ecosystems of such overlapping communities, (2) the reasons for the explosive growth of the Reddit cybersecurity community especially in terms of whether a discussion-based knowledge sharing site is more effective than the more traditional question and answer sites, and (3) to measure the level of expertise in the public domain (social question and answer sites), as a collective output.
